# Endoscope-Guided Interstitial Intensity-Modulated Brachytherapy and Intracavitary Brachytherapy as Boost Radiation for Primary Early T Stage Nasopharyngeal Carcinoma

**DOI:** 10.1371/journal.pone.0090048

**Published:** 2014-03-03

**Authors:** Xiang-Bo Wan, Rou Jiang, Fang-Yun Xie, Zhen-Yu Qi, Ai-Ju Li, Wei-Jun Ye, Yi-Jun Hua, Yu-Liang Zhu, Xiong Zou, Ling Guo, Hai-Qiang Mai, Xiang Guo, Ming-Huang Hong, Ming-Yuan Chen

**Affiliations:** 1 Department of Nasopharyngeal Carcinoma, Sun Yat-sen University Cancer Center, Guangzhou, P. R. China; 2 State Key Laboratory of Oncology in South China, Sun Yat-sen University Cancer Center, Guangzhou, P. R. China; 3 Department of Radiation Oncology, the Sixth Affiliated Hospital, Sun Yat-sen University, Guangzhou, P. R. China; 4 Department of Radiation Oncology, Sun Yat-sen University Cancer Center, Guangzhou, P. R. China; 5 Collaborative Innovation Center for Cancer Medicine, State Key Laboratory of Oncology in South China, Sun Yat-sen University Cancer Center; Guangzhou, P. R. China; University of California Davis, United States of America

## Abstract

**Background:**

Intracavitary brachytherapy (ICBT) is usually applied as boost radiotherapy for superficial residual of nasopharyngeal carcinoma (NPC) after primary extern-beam radiptherapy (ERT). Here, we evaluated the outcome of endoscope-guided interstitial intensity-modulated brachytherapy (IMBT) boost radiation for deep-seated residual NPC.

**Methodology/Principal Findings:**

Two hundred and thirteen patients with residual NPC who were salvaged with brachytherapy boost radiation during 2005–2009 were analyzed retrospectively. Among these patients, 171 patients had superficial residual NPC (≤1 cm below the nasopharyngeal epithelium) were treated with ICBT boost radiation, and interstitial IMBT boost radiation was delivered to 42 patients with deep-seated residual NPC (>1 cm below the nasopharyngeal epithelium). We found that IMBT boost subgroup had a higher ratio of T2b (81.0% VS 34.5%, *P*<0.001) and stage II (90.5% VS 61.4%, *P* = 0.001) than that of ICBT boost subgroup. The dosage of external-beam radiotherapy in the nasopharyngeal (63.0±3.8 VS 62.6±4.3 Gray (Gy), *P* = 0.67) and regional lymph nodes (55.8±5.0 VS 57.5±5.7 Gy, *P* = 0.11) was comparable in both groups. For brachytherapy, IMBT subgroup had a lower boost radiation dosage than ICBT subgroup (11.0±2.9 VS 14.8±3.2 Gy, *P*<0.01). Though the IMBT group had deeper residual tumors and received lower boost radiation dosages, both subgroups had the similar 5-year actuarial overall survival rate (IMBT VS ICBT group: 96.8% VS 93.6%, *P* = 0.87), progression-free survival rate (92.4% VS 86.5%, *P* = 0.41) and distant metastasis-free survival rate (94.9% VS 92.7%, *P* = 0.64). Moreover, IMBT boost radiation subgroup had a similar local (97.4% VS 94.4%, *P* = 0.57) and regional (95.0% VS 97.2%, *P* = 0.34) control to ICBT subgroup. The acute and late toxicities rates were comparable between the both subgroups.

**Conclusions/Significance:**

IMBT boost radiation may be a promising therapeutic selection for deep-seated residual NPC.

## Introduction

Radiotherapy is the primary and radical therapy for non-metastatic NPC [1]. Residual NPC carries an enhanced risk of locoregional recurrence and distant metastasis [2]. For patients with persistent NPC, the local control rate is nearly 40% [3]. To date, intracavitary brachytherapy remains the most effective salvage therapy for locally superficial residual NPC (≤1 cm below the nasopharyngeal epithelium) [4], [5]. For patients with T1-2 persistent NPC, the local relapse-free survival (LRFS) rate is 91.0–95.8% after ICBT boost radiation, whereas was 60.0–85.2% in patients without given ICBT boost [6], [7], [8]. Moreover, the overall survival (OS) rate can be elevated from 79.6% to 91.1% by adding the ICBT boost [9]. Importantly, ICBT had a limited dosage in paranasopharyngeal vital organs, and evidently reduced temporal lobe necrosis, cranial nerve palsy and endocrine dysfunction [4].

Despite advances in endoscopy, the narrow nasal cavity made the brachytherapy applicators can only be placed on the surface, rather than inserted into residual tumors [4]. Therefore, ICBT is empirically delivered to the superficial residual malignancies (T1-2a and part of T2b) [9], [10]. Maximizing local control is important to the radical treatment and the increasing of quality of life, since the residual tumor is the main source of locoregional recurrence and distant metastasis. In addition, the treatment outcomes of local and distant relapse are unsatisfactory, with a 5-year actuarial survival rates less than 37.8% [11], [12], [13], [14]. On the other hand, retreatment of locally recurrent NPC is associated with a high risk of complications, and up to 82.0% of patients develop re-irradiation related xerostomia, trismus, skin fibrosis, and deafness [4]. In light of the poor outcomes and high complication rates associated with retreatment, more effective salvage treatment should be developed to secure a higher local control and less toxicity.

In cervical carcinoma, interstitial intensity-modulated brachytherapy (IMBT) is always used to treat bulky tumors for better locoregional control, since conventional ICBT does not deliver an adequate and conformal dose to the tumor [15]. In a long-term study of interstitial IMBT in cervical carcinoma, though high-risk clinical target volume of mean 57 cm (3), the LRFS rate was still reached to 93.0% [16]. This cavity-based interstitial IMBT motivated us to address whether interstitial IMRT could achieve greater local control with less toxicity in patients with deep-seated residual NPC lesions.

Here, using endonasal endoscope guided applicator implanting method, we delivered IMBT boost radiation to deep-seated NPC residual lesions. Compared with patients receiving traditional ICBT boost radiation, the IMBT subset had a higher rate of T2b and was given a lower radiation dosage. Both groups had a comparable OS, progression-free survival (PFS), LRFS, regional relapse-free survival (RRFS) and distant metastasis-free survival (DMFS). Importantly, these two subsets had the similar acute and late toxicities. Our results suggested that the IMBT boost may be clinically useful to treat deep-seated NPC lesions.

## Materials and Methods

### Patients

From September 2005 to December 2009, 213 locally persistent NPC (T1-2) patients after the radical external beam radiotherapy at Sun Yat-sen University Cancer Center were recruited. The routine staging work-up consisted of a detailed clinical examination, fiberoptic nasopharyngoscopy or sinus endoscopy, MRI of the entire neck from the base of the skull, abdominal sonography, chest radiography, a complete blood count, and a biochemical profile. TNM stage was classified according to the 6th edition of the American Joint Commission on Cancer (AJCC) staging system [17]. Demographic features are summarized in Table 1. This study was approved by the Clinical Ethics Review Committee at Sun Yat-sen University Cancer Center. All of the patients had signed informed consent documents prior to participating in this study. And the subject of the photograph had signed written informed consent, as outlined in the PLOS consent form, to publication of his photograph.

**Table 1:** Patients characteristics.

Characteristics	ICBT (n = 171)		IMBT (n = 42)		*P* value
	No.	%	No.	%	
**Gender**					
Female	44	25.7	14	33.3	0.321
Male	127	74.3	28	66.7	
**Age**					
<43 years	76	44.4	21	50	0.517
≥43 years	95	55.6	21	50	
Pathological type	11	6.4	40	95.2	
WHO I/II	160	93.6	2	4.8	0.685
WHO III					
**T classification**					
T1	75	43.9	0	0	<0.001
T2a	37	21.6	8	19	
T2b	59	34.5	34	81	
**N classification**					
N0	85	49.7	17	40.5	0.398
N1	63	36.8	21	50	
N2	20	11.7	4	9.5	
N3	3	1.8	0	0	
**Overall stage**					
Stage I	43	25.1	0	0	0.001
Stage II	105	61.4	38	90.5	
Stage III	20	11.7	4	9.5	
Stage IV	3	1.8	0	0	
**Chemotherapy**					
Without	120	70.2	29	69	0.886
With	51	29.8	13	31	
**ERBT dose of nasopharyngeal**					
<62 Gy	111	64.9	25	59.5	0.515
≥62 Gy	60	35.1	17	40.5	
**ERBT dose of lymph node**					
<56 Gy	85	49.7	14	33.3	0.057
≥56 Gy	86	50.3	28	66.7	
**Interval of EBRT and boost**					
<3 days	60	35.1	10	23.8	0.163
≥3 days	111	64.9	32	76.2	
**Brachytherapy dose**					
<15 Gy	51	29.8	35	83.3	<0.001
≥15 Gy	120	70.2	7	16.7	
**Brachytherapy fractions**					
<4 fractions	56	32.7	10	23.8	0.262
≥4 fractions	115	67.3	32	76.2	
**Brachytherapy fractional dose**					
<4Gy	22	12.9	36	85.7	<0.001
≥4 Gy	149	87.1	6	14.3	
**Brachytherapy duration**					
<7 days	58	33.9	39	92.9	<0.001
≥7 days	113	66.1	3	7.1	
**Brachytherapy applicators**					
<3	167	97.7	18	42.9	<0.001
≥3	4	2.3	24	57.1	
**Accurate toxicities**					
RTOG grade 1–2	150	87.7	38	90.5	0.619
RTOG grade 3–4	21	12.3	4	9.5	
**Late toxicities**					
RTOG grade 1–2	163	95.3	41	97.6	0.507
RTOG grade 3–4	8	4.7	1	2.4	

Abbreviations: ICBT = intracavitary brachytherapy; IMBT = intensity-modulated brachytherapy; WHO = World Health Organization; RTOG = Radiation Therapy Oncology Group.

### External beam radiotherapy administration

External radiotherapy was delivered using two-dimensional conventional radiotherapy in 196 patients, and intensity-modulated radiotherapy (IMRT) in 17 cases. Radiation targets included the primary tumor (gross target volume of nasopharynx, GTVnx), positive cervical lymph nodes (GTVnd), and the head and neck regions with high risk of invasion (clinical target volumes, CTV) [4]. For two-dimensional conventional radiotherapy (2D-CRT), the total dose of radiotherapy was 50–72 Gy to the locally NPC and 50–70 Gy to the regional lymph nodes (2 Gy/fraction/day). For IMRT, the prescribed dose was 68–72 Gy/30–33 fractions to the GTVnx and 60–66 Gy/30–33 fractions to the GTVnd.The radiotherapy was administrated daily from Monday to Friday.

### Brachytherapy administration

**Figure 1:**
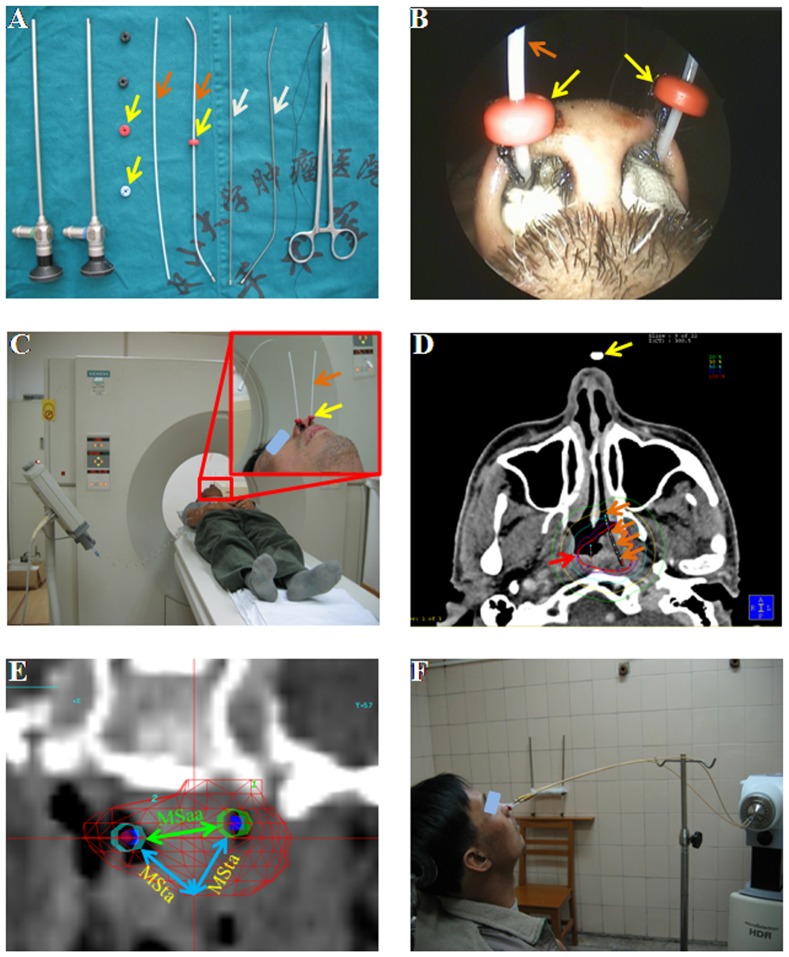
Endoscopic-guided IMBT boost administration process. (A) The instruments and applicators used in endoscopic-guided IMBT boost process. (B) The nasal outward view of applicators sewed to the nose wings. (C) CT scan of the tumor and applicators location. (D) CT images of tumor residue, applicators and isodose line. The red arrow indicated the 100% isodose curve covering the whole GTV. (E) The 3-dimensional reconstruction image of two applicators (outlined by hepta-prism) inserting into the nasopharyngeal residue (outlined by red fine grid lines) in coronal section. MSta was referred to the maximum spacing from the tumor margin to the nearest applicator (blue arrows), and MSaa was referred to minimum distance between two applicators (green arrow). (F) A representative case that was delivering the IMBT boost. The crimson arrow denoted the applicator outline, and white arrow displayed the 6F sharp Obturator with 0 and 30 degree angle. The yellow arrow indicated the Radio Opaque Button.

**Figure 2:**
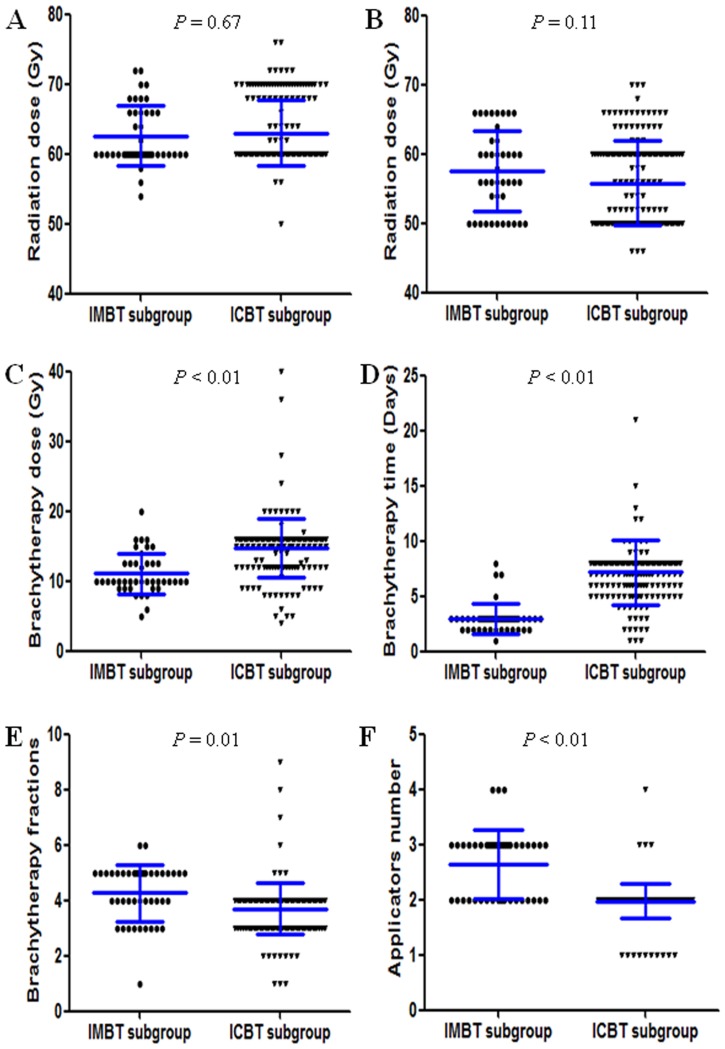
Brachytherapy related factors were compared in IMBT and ICBT boost subgroups. The external-beam radiotherapy doses in nasopharyngeal (A) and neck (B) zone were compared in IMBT and ICBT boost subgroups. The brachytherapy radiation dose (C), duration (D), fractions numbers (E) and applicator numbers (F) were compared in IMBT and ICBT boost subgroups.

**Figure 3:**
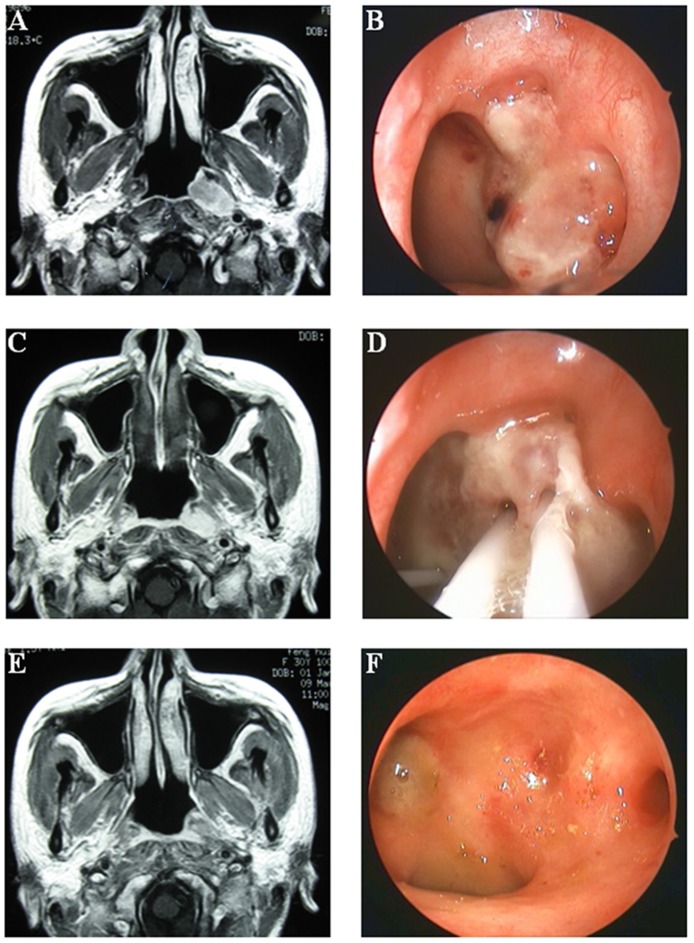
The representative images prior to, during and after the IMBT boost for T2b NPC. MRI view (A) and endoscopic image (B) of the nasopharyngeal carcinoma prior to the external beam radiotherapy. MRI view (C) and endoscopic image (D) of the residual nasopharyngeal carcinoma after the external beam radiotherapy. MRI view (E) and endoscopic image (F) of the nasopharyngeal after the external beam radiotherapy and IMBT boost radiation.

Boost radiation to the residual primary tumor sites would be withheld unless the biopsy remained positive at 10 or more weeks after external radiotherapy. The IMBT boost was delivered to the residual malignancy within the limited areas, including of the upper margin: the skull base bone; the lower margin: the inferior border of the second cervical vertebra; the anterior margin: the postnaris; the posterior margin: the prevertebral fascia; the anterolateral margin: the medial pterygoid plate; the posterolateral margin: the internal carotid artery. The IMBT boost radiation was administered as following: 1) the Obturators were bended to a suitable angle to fit the tumor configuration, and then inserted the 6F sharp Obturator into the applicator (OncoSmart ProGuide Needles) (Figure 1A); 2) under guidance of 4-mm rigid sinus endoscope (Karl-Storz, Germany), the obturator/applicator was inserted into the lidocaine anaesthetized residual tumor (Figure 1A and 1B) and scanned by computed tomography (Figure 1C); 3) after pulling out the obturator, the applicators were placed using the Radio Opaque Button on the nose wings (Figure 1B and 1C).

The following 3 obturator/applicator implantation principles were adhered: 1) two to four applicators were used for further dosage optimization; (2) the obturator/applicator angle was less than 30 degrees to avoid obstruction of the radiation source (^192^Ir); (3) the obturator/applicator implantation pathway deviated from internal carotid artery to avoid an accidental injury.

Prior to the brachytherapy boost, CT simulation was performed extending from the sellar base to the second cervical vertebra with a slice thickness of 2 mm (Figure 1C). The accuracy of implantation was assessed by evaluating the maximum distance from the tumor margin to the nearest applicator (MDta), and maximum distance between nearby applicators (MDaa). The implantation was acceptable when MDta was ≤1.0 cm and MDaa ≤2.0 cm. Moreover, the tumor target zone and sensitive organs were delineated on each CT slice using the PLATO brachytherapy planning system (Version 14.2.6, Nucletron, Veenendaal, Netherlands). In the planning process, the dosage distribution was optimized to ensure GTV might be covered completely, while the nearby important organs were exposed to less than 20–50% of maximum dosage (Figure 1D and 1E).

The MicroSelectron ^192^Ir, with the 0.9 mm total diameter and 3.6 mm length, High-Dose-Rate Brachytherapy System (Nucletron, Netherlands) was used to deliver ICBT and IMBT boost radiation (Figure 1F). For ICBT boost, the applicator would be pulled out after each radiation fraction, whereas would be sustained to all fractions in IMBT boost to avoid repeated implanting. The ICBT boost fraction of 3–5 Gy was delivered daily, and IMBT boost fraction of 2–3 Gy was delivered twice daily at 6–8 hours interval.

### Statistical analysis

The survival rate was calculated using Kaplan-Meier analysis, and the differences between groups were compared using the log-rank test. All events were measured from the date of brachytherapy. Additionally, Cox multivariate model was used to evaluate the prognostic effect of brachytherapy boost radiation on OS, PFS, LRFS, RRFS, and DMFS. A two-tailed *P*<0.05 was considered statistically significant. Statistical analysis was performed using SPSS 17.0 (SPSS, Inc., Chicago, IL).

## Results

### Patient demography

As shown in Table 1, the rates of T2b (81.0% vs 34.5%, *P*<0.001) and stage II (90.5% vs 61.4%, *P* = 0.001) were higher in the IMBT boost group than that of ICBT boost group. Conversely, the rate of T1-T2a was higher in the ICBT boost group than that of IMBT boost group (65.5% vs 19.0%, *P*<0.001). However, the distribution of N classification (ICBT VS IMBT, *P* = 0.398) was comparable between these two subgroups. Furthermore, no statistical difference was observed in gender, age, pathological type and chemotherapy status for both subgroups.

### Radiotherapy administration

Prior to brachytherapy boost radiation, the median dosage of external beam radiation that delivered to the nasopharynx and regional lymph nodes was 62.0 (range: 50.0–76.0) Gy and 56.0 (range: 46.0–70.0) Gy, respectively (Table 1). Both groups received a comparable dosage in the nasopharynx (ICBT group vs IMBT group: 63.0±3.8 vs 62.6±4.3 Gy, *P* = 0.67, Table 1, Figure 2A) and regional lymph nodes (ICBT group vs IMBT group: 55.8±5.0 vs 57.5±5.7 Gy, *P* = 0.11, Table 1, Figure 2B). For brachytherapy, the total dosage (11.0±2.9 vs 14.8±3.2 Gy, *P*<0.001, Table 1 and Figure 2C), duration (3.0±1.4 vs 7.1±2.1 days, *P*<0.001, Table 1 and Figure 2D), and number of fractions (4.3±1.0 VS 3.7±0.9 fractions, *P*<0.01 and Figure 2E) of IMBT boost radiation were lower, shorter, and larger than that of ICBT boost radiation, respectively.

Prior to brachytherapy administration, two applicators (2.0±0.3 applicators) were mounted to deliver brachytherapy in 97.7% (167/171) of the ICBT boost group. To obtain a favorable dosage distribution, more than two applicators (2.6±0.6 applicators) were implanted to deliver brachytherapy in 57.1% (24/42) of the IMBT boost group (*P*<0.001, Table 1 and Figure 2F). The applicators were all successfully implanted without severe surgical complications. Importantly, similar acute and late toxicities were observed in both groups (*P*>0.05, Table 1).

### Clinical outcome

**Figure 4:**
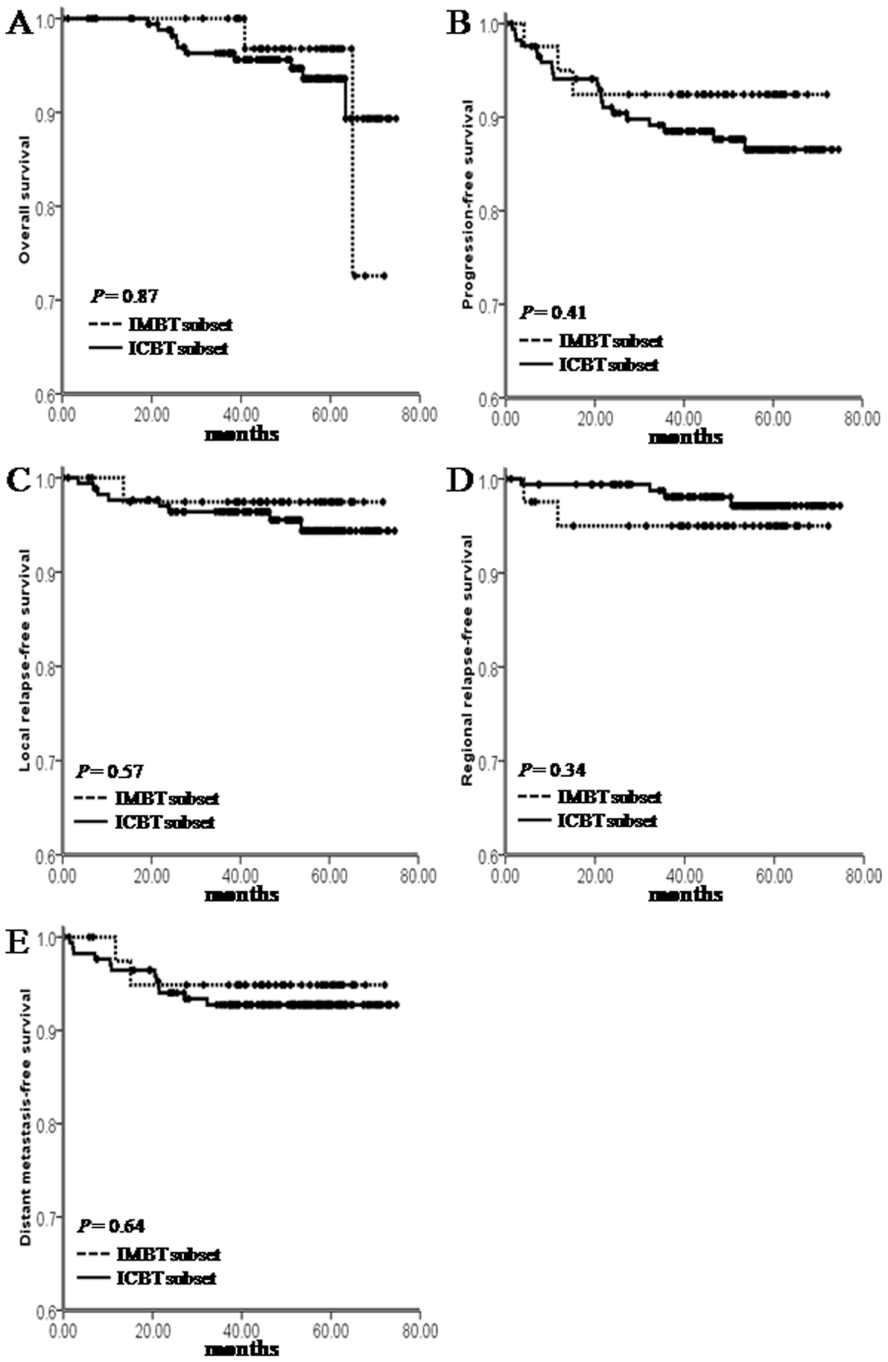
Kaplan-Meier estimated of OS, PFS, LRFS, RRFS and DMFS in ICBT and IMBT boost radiation groups.

The residual tumors in both groups were all complete regressed within 3 month after the brachytherapy boost radiation (Figure 3). For the overall patients, the follow-up duration was 0.17 to 74.70 months (median, 53.03 months). Additionally, 6 (3.5%), 2 (1.2%), 10 (5.8%), and 10 (5.8%) patients respectively developed in situ recurrence, regional lymph failure, distant metastasis, and died at the latest censored time in the ICBT group, and 1 (2.4%), 2 (4.8%), 2 (4.8%), and 2 patients (4.8%) respectively developed local failure, regional failure, distant failure, and died at the latest censored time in IMBT group. Importantly, the survival analysis showed that, though IMBT group had a higher T2b rate and lower brachytherapy dose, both groups had similar OS (ICBT vs IMBT, 93.6% vs 96.8%, *P* = 0.87), PFS (86.5% vs 92.4%, *P* = 0.41), LRFS (94.4% vs 97.4%, *P* = 0.57), RRFS (97.2% vs 95.0%, *P* = 0.34), and DMFS rate (92.7% vs 94.9%, *P* = 0.64) (Figure 4).

### Multivariate analysis

Cox regression analysis further proved that, though IMBT boost subgroup had a higher rates of T2b and TNM stage (Table 1), both subsets had the similar risk to death (*P* = 0.61), disease progression (*P* = 0.27), local (*P* = 0.43) as well as regional (*P* = 0.44) relapse, and distant metastasis (*P* = 0.50). Moreover, gender, age, pathological type, T stage, N stage, and TNM stage had no significant prognostic effect to the outcome of IMBT and ICBT boost radiation (Table 2).

**Table 2:** Results of multivariate Cox proportional-hazards analysis.

Factors	Death		Disease progression		Local relapse		Regional lymph relapse		Distant metastasis	
	HR (95%CI)	*P* value	HR (95%CI)	*P* value	HR (95%CI)	*P* value	HR (95%CI)	*P* value	HR (95%CI)	*P* value
Gender (male VS female)	0.630 (0.157–2.526)	0.514	0.555 (0.204–1.510)	0.249	0.256 (0.031–2.101)	0.204	--	0.976	0.870 (0.269–2.814)	0.816
Age (43 VS ≥43)	2.361 (0.660–8.448)	0.186	0.703 (0.310–1.592)	0.398	0.600 (0.156–2.308)	0.457	0.978 (0.166–5.766)	0.980	0.671 (0.229–1.962)	0.466
Pathological type (I VS II-III)	0.302 (0.032–2.876)	0.298	0.545 (0.156–1.901)	0.341	0.587 (0.067–5.150)	0.631	0.584 (0.054–6.345)	0.659	1.068 (0.136–8.408)	0.950
T stage (T_1-2a_ VS T_2b)_	1.619 (0.324–8.098)	0.558	1.648 (0.627–4.330)	0.311	2.128 (0.414–10.950)	0.366	1.832 (0.178–18.879)	0.611	1.803 (0.472–6.891)	0.389
N stage (N_0-1_ VS N_2-3)_	2.569*10^6^ (0.001–1.260*10^6^)	0.929	1.467 (0.554–3.881)	0.440	1.733 (0.373–8.060)	0.483	1.199 (0.162–8.897)	0.859	1.495 (0.395–5.665)	0.554
Branchytherapy types (IMBT VS ICBT)	0.808 (0.359–1.820)	0.607	0.703 (0.375–1.318)	0.272	0.650 (0.223–1.896)	0.430	1.445 (0.572–3.649)	0.437	0.764 (0.350–1.667)	0.499

Abbreviation: HR = hazard ratio; IMBT = intensity-modulated brachytherapy; ICBT = Intracavitary brachytherapy.

## Discussion

Branchytherapy has the advantage of focusing the radiation dose within a 1 cm diameter treatment volume and quickly disappearing thereafter [18]. For NPC, this feature maximizes the radiation dose delivered to the residual lesions, and minimizes the exposure of normal adjacent organs to irradiation [9]. Therefore, the 1 cm treatment distance was always used in ICBT boost treatment for superficial NPC residual lesions (T1-2a) [9]. Wang et al. reported that external beam radiotherapy plus brachytherapy boost radiation (compared with external beam radiotherapy alone) increased a 39% (93% VS 54%) of 5-year local control benefit for T1 NPC residual lesions [6]. We and others studies found that ICBT might reduce 7.6% (14.1% VS 21.7%) 5-year disease-specific mortality for the recurrent T1-2a NPC residue [19], [20]. Though had the limitation of 1 cm treatment distance, brachytherapy boost radiation had been reported be helpful for treating T2b NPC residual lesions with diameter larger than 1 cm [21], [22]. In a 34 patients small-size study, Leung et al. found that adding ICBT boost radiation to external beam radiotherapy significantly improved 5-year LRFS, PFS, and OS rates for persistent T2b NPC [21]. Importantly, Leung et al. also noticed that, though the 5-year LRFS rate (96.9%) was encouraging, it remained unknown whether the residual tumor volume extended beyond the prescribed depth of the brachytherapy [21]. These results suggested that the role of conventional ICBT boost radiation was still debatable for T2b persistent lesions. For the underlying mechanism, it is reasonable that the narrow nasal cavity greatly limit the accurate positioning of the applicators in ICBT. Moreover, the applicators would be pulled out after the completion of each ICBT and be mounted prior to the next ICBT under the blind condition. These limitations leads to that the applicators placed on the surface of persistent NPC lesions are unstable and prone to float within the nasal cavity. Therefore, for the large and deep persistent T2b NPC lesions, unstable applicators would not be enough to secure an adequate ICBT boost.

Previously, we reported that endoscopically implanted applicators were effective to deliver the brachytherapy boost for recurrent T1-2a NPC residual lesions [20]. We inferred that the applicator implantation technique might also be used to the treatment of primary NPC residues. More importantly, the applicators were accurately placed within the target area, and were positioned at the same point during the whole IMBT process. Therefore, the dosage distribution was easily to be optimized, and the treatment efficacy of large or deep residual lesions might be greatly improved in IMBT. Indeed, we confirmed that, though the T2b rate was higher and boost dose was lower in the IMBT group (Table 1 and Figure 1C), the 5-year local control rate (97.4% vs 94.4%, *P*>0.05), overall survival rate (96.8% vs 93.6%, *P*>0.05), and toxicities were comparable between ICBT and IMBT subgroups. These dose and survival advantages suggested that the IMBT boost might be a promising selection for deep-seated NPC residual lesions.

Recently, the high activity micro ^192^Ir source stepping system was used as the basis for an individualized 3-dimensional (3D) brachytherapy planning system [23]. The Nucletron branchytherapy 3D planning system, for example, arranges applicators using a modified Paris model, and further optimizes the IMBT dosage to conform to the tumor shape [24], [25]. In this study, the Nucletron branchytherapy 3D planning system was used to deliver the IMBT dosage. We found that the 100% isodose covered the contours of the primary nasopharyngeal and parapharyngeal carcinoma completely, and the dosage distribution was satisfactory. Furthermore, combined with the external beam radiotherapy, the total dosage was easily elevated to 70–80 Gy for all patients. However, we also realized to that, due to the short treatment distance of branchytherapy, this novel IMBT approach was limited to selected patients rather than to all T2b-4 patients. Additionally, IMBT is not suitable for bulky residual lesions since the nasal cavity was too narrow to implant more than 4 applicators, which might be the minimal number to obtain a satisfactory dosage distribution for bulky cervical carcinoma. As mentioned in a previous study, if the tumor is bulky, IMBT dosage optimization and distribution might be compromised, leading to a poor local control rate for persistent lesions [21]. Thus, IMBT boost radiation might be a promising therapeutic technique for selective ≥ T2a NPC residual lesions.

In general, using endoscopic guidance to deliver interstitial IMBT boost radiation to T2a-2b NPC residual lesions, the radiation dose can readily be increased to radical level with less complication. Interstitial IMBT boost under endoscopic guidance might be a safe and effective complement to external radiotherapy for selected primary NPC residual lesions.
